# Supplementary material for: Association between sarcopenia level and metabolic syndrome
Source: PLoS One. 2021 Mar 19;16(3):e0248856. doi: 10.1371/journal.pone.0248856 (PMC7978348; doi:10.1371/journal.pone.0248856)
Supplement: Attachment — Submitted filename: Response to reviewers.docx [file pone.0248856.s002.docx]

**Response Letter**

Editors, PLOS ONE

Thank you for allowing the revision of our manuscript: ID PONE-D-20-39066 entitled “Association between sarcopenia level and metabolic syndrome” We revised our manuscript in accordance with the reviewers’ suggestions. Our responses to the comments are as follows.

**Reviewer #1:**

This article elucidated the relationship between the prevalence of metabolic syndrome (MetS) and the degree of muscle loss. I read very interestingly. I have some questions and propose some revisions to authors.

**Comment 1>**
(1) I feel that Reference 1 and 2 are little bit old.

*Reply: Thank you for your valuable comments. We added* *newer references.*

**Comment 2>**
(2) You mentioned the relationship between sarcopenia and gallbladder polyps in Introduction part. Is this sentence necessary? I think the clinical importance of gallbladder polyps are not equal to other diseases, such as cancer, diabetes, which you mentioned.

*Reply: Thank you for your valuable comments. Based on your comments, we eliminated gallbladder polyps from the sentence which you commented on. We revised our manuscript as follows.*

*MetS increases the risk of diabetes mellitus (DM), CVD, chronic liver disease and hepatocellular carcinoma, other cancers, and mortality.*

**Comment 3>**
(3) You cited Reference 28 as the definition of MetS. I think your definition is as same as the NCEP ATP-III, so you should add more suitable reference.

*Reply: Thank you for your valuable comments. We added a suitable reference as you commented.*

**Comment 4>**
(4) In statistical analysis, I have three questions. First, why did you adopt C-reactive protein level as variable? Second, why did not you adopt smoking -/+ or alcohol intake -/+ as variables? I think the lean is also related to sarcopenia and Mets. Why did not you adopt body mass index as variables? In addition, I would like to know the percentage of obesity and lean participants in Table 1, if possible.

*Reply: Thank you for your valuable comments. We revised our manuscript as follows with suitable references.*

- *We adopted CRP as a variable as previous studies have shown the association between CRP and metabolic syndrome.*

*According to your comments, we added another model adjusted with smoking and alcohol intake. We revised Table 2, 4, 5.*

*We intended to analyze our data from the aspect of MetS component. Thus, we adopted obesity as a binary variable instead of using BMI as a numerical variable.*

*We added the percentage of obesity, overweight, normal BMI and lean (underweight) participants in Table 1.*

**Comment 5>**
(5) The characteristics of patients with sarcopenia are heterogenous. Q1 group contained older, heavier, and less ASM and ASM (%) participants. Because you analyzed using many variables, such as age, sex, and obesity, I can accept your opinion. I would like to know results of subgroup analysis according to lean in Table 3.

*Reply: Thank you for your valuable comments. We performed subgroup analysis according to lean (underweight) in Table 3. We modified Table 3 according to your comments.*

**Reviewer #2:**

**Comment 1>**

1. This study is a cross-sectional and single-centered retrospective research. Thus it was difficult to assess the causal relationship between sarcopenia and MetS. However, the researchers have the opportunity to study the causal relationship by the repeated health checkup(n=4621). I would like to be showed the corresponding results.

*Reply: Thank you for your valuable comments. As you mentioned, it seems that causal relationship may be assessed by analyzing the data of subjects who underwent repeated health checkup. However, in the current study, we intended a cross-sectional study. We indeed have a plan to perform another study in the next step with the data of subjects who underwent repeated health checkup. As of now, it’s a pity that we can’t show you the data you want, but we’ll be able to show you when the next paper is completed. We sincerely ask for your kind understanding.*

**Comment 2>**
2. Some typos should be corrected, such as P13 TG≥ 150 g/dL

*Reply: Thank you for your valuable comments. We revised our manuscript as follows.*

TG ≥ 150 mg/dL or the use of medication

Thank you again for your insightful advice.

Yours sincerely,

Ji Bong Jeong, MD, PhD

Associate Professor

Department of Internal Medicine

Seoul Metropolitan Government Seoul National University Boramae Medical Center

20 Boramae-ro 5-gil, Dongjak-gu

Seoul 07061, Republic of Korea

Phone: +82-2-870-2222

Fax: +82-2-870-3863

E-mail: jibjeong@gmail.com
