# Supplementary material for: Association between sarcopenia level and metabolic syndrome
Source: PLoS One. 2021 Mar 19;16(3):e0248856. doi: 10.1371/journal.pone.0248856 (PMC7978348; doi:10.1371/journal.pone.0248856)
Supplement: Attachment — Submitted filename: Response to reviewers_2.docx [file pone.0248856.s003.docx]

**Response Letter**

Editors, PLOS ONE

Thank you for allowing the revision of our manuscript: ID PONE-D-20-39066 entitled “Association between sarcopenia level and metabolic syndrome” We revised our manuscript in accordance with the reviewers’ suggestions. Our responses to the comments are as follows.

**Reviewer #1:**

Thank you very much for answering my questions. I understood your reply well. I think this research article is very valuable. Thanks to authors and academic editor, I was able to have an important experience. As you plan to perform another study, I hope your next research will also be successful.

**Comment 1>**
(1) Thank you for your adding references.

**Comment 2>**
(2) After this, the topic of polyps was not mentioned, so I think the text became clear and concise. Thank you.

**Comment 3>**
(3) Thank you very much for adding a reference.

**Comment 4>**
(4) Thank you very much for telling me why you adopt CRP as a variable.
I have known the close relation between high-sensitivity CRP and chronic inflammation (as Ref 41 mentioned).
This time I understand the relation between CRP (not hs CRP) and metabolic syndrome (Ref40).
In addition, I appreciate your revising tables and adding the information of lean.
I understand your intention.

**Comment 5>**
(5) Thank you for conducting sub group analysis. I think the result of lean is also consistent with the sentence “the association between sarcopenia and MetS seemed more prominent in participants with low visceral fat or in non-obese participants”.

*Reply: Thank you for your valuable comments.*

**Reviewer #2:**

**Comment 1>**

1. Still it was difficult to assess the causal relationship between sarcopenia and MetS. Therefore, the conclusion should be revised. If the author think that sarcopenia by BIA is independently associated with the risk of MetS and has a dose-response relationship, the repeated health checkup would still be needed. I suggest modification of conclusion.

*Reply: Thank you for your valuable comments. Based on your comments, we revised our manuscript as follows.*

In conclusion, our study demonstrated that sarcopenia by BIA is independently associated with the risk of MetS and with a dose-response relationship. Future studies that assess causal relationship between sarcopenia and MetS are needed using the data of subjects who underwent repeated health checkup. By measuring sarcopenia using BIA, the risk of MetS can be assessed easily, safely, and cost-efficiently. BIA can be used as an easy, useful, and important guide to identify participants with the risk of MetS.

Thank you again for your insightful advice.

Yours sincerely,

Ji Bong Jeong, MD, PhD

Associate Professor

Department of Internal Medicine

Seoul Metropolitan Government Seoul National University Boramae Medical Center

20 Boramae-ro 5-gil, Dongjak-gu

Seoul 07061, Republic of Korea

Phone: +82-2-870-2222

Fax: +82-2-870-3863

E-mail: jibjeong@gmail.com
